# The Chromatin Organization Close to SNP rs12913832, Involved in Eye Color Variation, Is Evolutionary Conserved in Vertebrates

**DOI:** 10.3390/ijms25126602

**Published:** 2024-06-15

**Authors:** Desiree Brancato, Francesca Bruno, Elvira Coniglio, Valentina Sturiale, Salvatore Saccone, Concetta Federico

**Affiliations:** Department Biological, Geological and Environmental Sciences, University of Catania, Via Androne 81, 95124 Catania, Italy; desiree.brancato@phd.unict.it (D.B.); francesca.bruno@unict.it (F.B.); elvira_con99@icloud.com (E.C.); valentina.sturiale@phd.unict.it (V.S.); concetta.federico@unict.it (C.F.)

**Keywords:** genome organization, chromatin loops, eye color, externally visible characteristics, genetic polymorphisms, *OCA2* gene, *HERC2* gene, SNP rs12913832, in situ hybridization

## Abstract

The most significant genetic influence on eye color pigmentation is attributed to the intronic SNP rs12913832 in the *HERC2* gene, which interacts with the promoter region of the contiguous *OCA2* gene. This interaction, through the formation of a chromatin loop, modulates the transcriptional activity of *OCA2*, directly affecting eye color pigmentation. Recent advancements in technology have elucidated the precise spatial organization of the genome within the cell nucleus, with chromatin architecture playing a pivotal role in regulating various genome functions. In this study, we investigated the organization of the chromatin close to the *HERC2/OCA2* locus in human lymphocyte nuclei using fluorescence in situ hybridization (FISH) and high-throughput chromosome conformation capture (Hi-C) data. The 3 Mb of genomic DNA that belonged to the chromosomal region 15q12-q13.1 revealed the presence of three contiguous chromatin loops, which exhibited a different level of compaction depending on the presence of the A or G allele in the SNP rs12913832. Moreover, the analysis of the genomic organization of the genes has demonstrated that this chromosomal region is evolutionarily highly conserved, as evidenced by the analysis of syntenic regions in species from other Vertebrate classes. Thus, the role of rs12913832 variant is relevant not only in determining the transcriptional activation of the *OCA2* gene but also in the chromatin compaction of a larger region, underscoring the critical role of chromatin organization in the proper regulation of the involved genes. It is crucial to consider the broader implications of this finding, especially regarding the potential regulatory role of similar polymorphisms located within intronic regions, which do not influence the same gene by modulating the splicing process, but they regulate the expression of adjacent genes. Therefore, caution should be exercised when utilizing whole-exome sequencing for diagnostic purposes, as intron sequences may provide valuable gene regulation information on the region where they reside. Thus, future research efforts should also be directed towards gaining a deeper understanding of the precise mechanisms underlying the role and mode of action of intronic SNPs in chromatin loop organization and transcriptional regulation.

## 1. Introduction

Human pigmentation, including skin, eye, and hair color, is influenced by a combination of genetic and environmental factors [1,2]. Among these, genetic variation, particularly single-nucleotide polymorphisms (*SNPs*), plays a significant role [3,4]. Genome-wide association studies (GWASs) using high-density genotyping arrays have identified more than 11,000 SNPs associated with numerous physiological phenotypes [5]. By understanding the genetic basis of pigmentation, researchers have developed predictive models that can estimate an individual’s likely pigmentation traits based on his/her genetic profile [6,7]. This can have implications in forensic, anthropology, and evolutionary biology. In fact, DNA analyses from crime scenes can be used to predict the likely appearance of an unknown individual, aiding in criminal investigations [8]. Similarly, in anthropology, these *SNPs* can provide insights into ancient populations and human migration patterns based on inferred phenotypic traits [9].

While GWASs have advanced our understanding of pigmentation genetics, there are still gaps in knowledge, including understanding rare variants, gene–environment interactions, and epigenetic influences. In fact, the vast majority (more than 80%) of GWASs tag SNPs was located in intergenic or intronic regions, and the effect of noncoding SNPs is not directed at protein sequence variation; instead, they are more likely to influence gene regulation [10]. Additionally, DNA variants related to eye color are largely located in non-coding regions of the involved genes, and only some of these constitute potential regulatory elements for distal genes. Therefore, more studies are needed to understand how other non-coding DNA markers can carry the same pigmentation information as coding ones, with the goal of obtaining higher levels of prediction, especially for intermediate colors [11]. The *HERC2/OCA2* locus currently exerts the strongest genetic influence on eye color, where an intronic SNP, rs12913832, located within the *HERC2* gene, interacts with the *OCA2* promoter via chromatin looping [12,13]. This locus allows for the accurate prediction of blue-brown eye color [14,15], but intermediate eye color cannot yet be genetically predicted [16].

The intricate mechanisms governing gene expression control, particularly concerning DNA packaging, remain incompletely understood. In all eukaryotes, the genome is organized within the nuclear space, facilitating gene activation, transcriptional regulation, and the irreversible gene silencing. Achieving these functions necessitates a well-organized nuclear structure, with chromatin architecture pivotal in maintaining cellular health. Alongside the DNA sequence and defined chromatin structure, the spatial positioning of specific regions within the cell nucleus constitutes genome organization, sometimes referred to as spatial epigenetics [17,18]. Genome organization encompasses the spatial arrangement of individual chromosomes, single chromosomal bands, clusters of genes, and individual gene loci within cell nuclei, whereby they reside in nonrandom locations [19,20,21,22].

In the last two decades, radial chromosome positioning has been observed in all animal cells, as demonstrated by several studies [23,24,25,26]. Overall, studies on nuclear chromatin compartmentalization have shown an association between the position of a locus in the nucleus and some of its functional features, such as gene expression. Multiple studies have demonstrated that the inner part of the nucleus contains the majority of transcriptionally active chromatin, while the nuclear periphery is generally characterized by the presence of transcriptionally inactive heterochromatin [27,28,29,30,31,32,33,34]. Furthermore, genes located on individual chromosomes are also radially and nonrandomly positioned within cell nuclei. Therefore, chromosomal territory organization in the nucleus and chromatin architecture are evolutionarily highly conserved to ensure the proper functioning of the genome. Any repositioning can result in ectopic alterations in various functional properties, including the transcriptional control of genes [35,36,37,38,39].

Fluorescence in situ hybridization (FISH) utilizing various types of probes, from single loci to entire chromosomes, has revealed a general and conserved higher-order chromatin organization within the nucleus [17,40]. Additionally, FISH experiments using genomic DNA with specific GC-levels, or DNA obtained from chromosomal bands with varying GC-levels, have further deepened our understanding of genome organization within the nucleus [21,39,40,41,42]. More recently, the Hi-C method has significantly advanced our understanding of nuclear chromatin architecture by revealing the spatial proximity of individual genomic sequences through the cross-linking of chromatin regions that are joined or very close to one another [43,44,45]. This technique allows for the determination of both intra- and inter-chromosomal contacts, with all interactions between chromosomal regions being sequenced and considered when constructing computer-generated maps of the genome [46]. Hi-C results have demonstrated that the genome is organized into specific structures known as “topologically associated domains” (TADs), which remain stable across numerous cell divisions, exhibit invariance across different cell types, and are evolutionarily conserved among related species [42,47]. TADs are considered the fundamental units of chromosome folding, wherein chromosomes occupy distinct structural compartments referred to as A and B. Compartment A is correlated with gene-dense regions and is highly enriched with open chromatin, while compartment B is associated with closed chromatin [45]. Each corresponds to the previously identified gene-dense (compartment A) and gene-poor (compartment B) chromosomal regions, observed by in situ hybridization, occupying the more internal and the more peripheral nuclear compartment, respectively [21,48,49]. TADs organize themselves into autonomous domains of the genome, exerting significant roles in transcriptional regulation, DNA replication, and other processes involving chromatin organization [50,51]. Consequently, the organization of the genome into defined structural domains is a crucial feature for genome functionality, as the previously described in human pigmentation studies related to hair or eye colors [13,52,53,54,55].

The intron 86 of the *HERC2* gene, where the rs12913832 SNP is situated, is highly conserved in mammals, and contains a consensus binding site for the helicase-like transcription factor (HLTF) [56]. Specifically, this SNP functions as an enhancer that regulates *OCA2* transcription, with transcription factors HLTF, LEF1, and MITF binding to the rs12913832 site. The A allele allows for the transcriptional activation of the *OCA2* gene via the formation of a loop, whereas the G allele does not [13]. Our present investigation aims to clarify whether this SNP plays a role not only in the regulation of the *OCA2* gene but also in the chromatin compaction of a larger chromosomal region. Therefore, to obtain information on this, we analyzed the chromatin organization of 3 MB of genomic DNA from the chromosomal region 15q12-q13.1 in the nuclei of human lymphocytes from individuals with different rs12913832 genotypes. This was performed using FISH and Hi-C methods, allowing us to obtain data from two orthogonal approaches to elucidate the organization of the aforementioned region. Furthermore, we analyzed the evolutionary conservation of the organization of the genes located in this chromosomal region in different Vertebrate species.

## 2. Results

### 2.1. Genomic Features of the 15q12-q13.1 Chromosome Band

**Figure 1:**
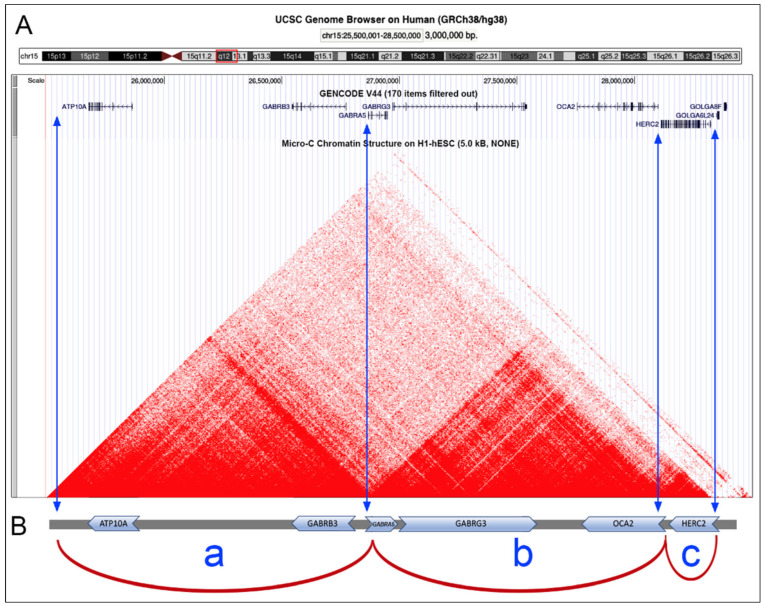
Genomic organization of the *HERC2/OCA2* locus. (**A**) Features of the 3 Mb genomic region of the human chromosomal bands 15q12-q13.1 (highlighted by the red box in the upper ideogram): genes with their transcriptional orientation and the intron/exon organization and contact map (visualized by a Hi-C heat map) on H1-hESC cells. Image from the UCSC Genome Browser (https://genome.ucsc.edu, accessed on 5 March 2024). (**B**) Schematic representation of the three chromatin loops (a, b, c) identified in this region, taking into consideration the above contact map. The vertical blue arrows indicate the loop transition sites.

The *HERC2* and *OCA2* genes are situated adjacent one to each other in a head-to-tail configuration, with transcription occurring in the same direction towards the centromeric part (Figure 1). In addition to these genes, the 3 Mb region corresponding to the 15q12-q13.1 bands also encompasses the genes *GABRG3*, *GABRA5*, *GABRB3*, and *ATP10A* [57,58,59,60]. Among these, *GABRG3* and *GABRA5* are oriented oppositely to *OCA2*/*HERC2*, while *GABRB3* and *ATP10A* share the same orientation. The *ATP10A* gene encodes for the ATPase Phospholipid Transporting 10A belonging to the family of P-type cation transport ATPases. This gene is maternally expressed and maps within the most common interval of deletion responsible for Angelman syndrome. *GABRB3* encodes for the Gamma-Aminobutyric Acid Type A Receptor Subunit Beta3, a member of the ligand-gated ionic channel family. The encoded protein serves as the receptor for gamma-aminobutyric acid, a major inhibitory neurotransmitter of the mammalian nervous system. It is located on the long arm of chromosome 15 in a cluster with two other genes (*GABRA5* and *GABRG3)* encoding related subunits of the family and may be associated with the pathogenesis of several disorders, including Angelman and Prader–Willi syndromes [61].

The contact map of this region, as defined by the heat map of Hi-C data in the H1-hESC cells, clearly highlights the presence of three chromatin loops, one comprising the *ATP10A* and *GABRB3* genes; the second comprising the *GABRA5*, *GABRB3*, and *OCA2* genes; and the third, the smaller one, only containing the *HERC2* gene (Figure 1). The border between the loops containing the *OCA2* and *HERC2* genes comprises the intergenic region between these two genes, thus with the regulatory sequences of *OCA2* located in this region and the SNP rs12913832, located in the 86th intron of *HERC2*, very close to this loop border.

**Figure 2:**
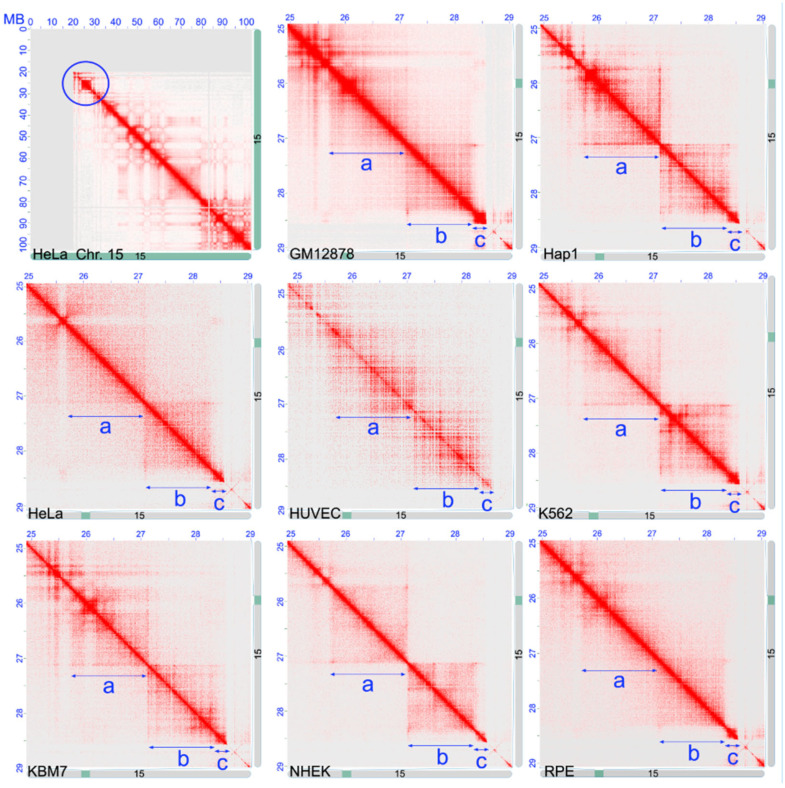
Contact maps of the chromosomal band 15q12-q13.1 in different cell types. (**Upper left**) Heat map (resolution 500 kb) of the entire chromosome 15 in the HeLa cell line. The blue circle indicates the chromosomal region further analyzed in a more detail. The other images show the contact map (resolution 5 kb) of the 15q12-q13.1 region observed in different cell types: *GM12878*, human B-lymphoblastoids; *Hap1*, near-haploid human chronic myelogenous leukemia; *HeLa*, human cervical carcinoma; *HUVEC*, human umbilical vein endothelial; *K562*: human erythroleukemia; *KBM7*, near-haploid human myelogenous leukemia; *NHEK*, normal human epidermal keratinocytes; *RPE1*, human retinal pigmented epithelial. The blue arrows and the small letters a, b, and c indicate the position of the three chromatin loops shown in Figure 1. Data from Ref. [62] (Hap1), [47] (hESC), [63] (RPE1), and [64] (the other cell lines).

The heat maps of the chromosomal region 15q12-q13.1, at a resolution of 5 kb, were obtained in nine different human cell lines. These showed a high level of similarity, as indicated by the presence of the three chromatin loops (Figure 2) with a similar size to those identified in hESC (Figure 1).

### 2.2. Organization of the Human 15q12 Region in the Cell Nucleus

**Figure 3:**
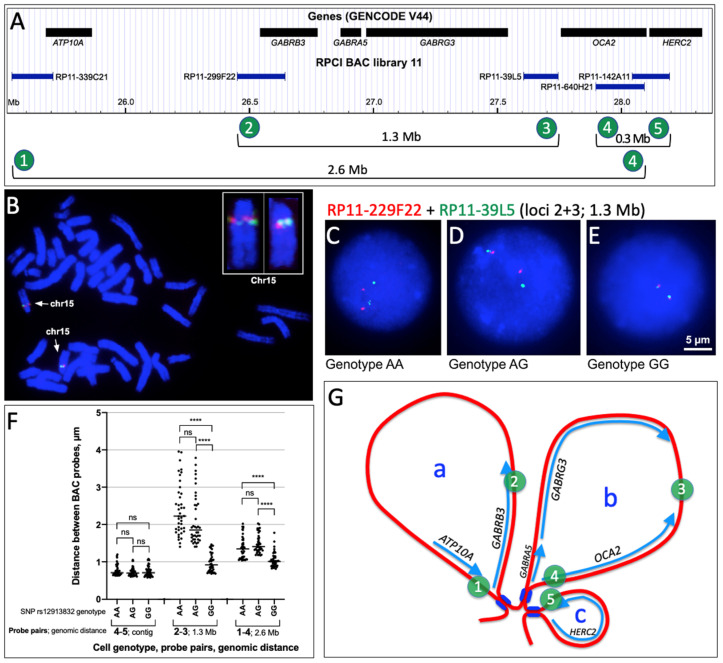
Variation in the *HERC2/OCA2* loop organization depending on the rs12913832 genotype. (**A**) Position of the BAC probes used in the fluorescence in situ hybridization (FISH). The numbers 1 to 5 represent the five BAC probes here used. The genomic distances in Mb (Mega base pairs) between the probe pairs #1#4, #2#3, and #4#5 are indicated. (**B**) Representative metaphase plate from lymphocyte cells showing the location by dual color FISH of a probe pair, as example, used in the present work. The red (rhodamine) and green (fluoresceine) spots show the location of the probes in the chromosome 15 pair (highlighted in the upper right part). Chromosomes were stained with DAPI (blue). (**C**–**E**) Examples of FISH results in the cell nuclei to measure the physical distance of each pair of probes. The probe pair #2#3 (RP11-299F22^FITC^ and RP11-39L5^RHOD^) was hybridized in lymphocyte cells with AA, AG, and GG genotype for the rs12913832 SNP. Only pair signals clearly belonging to the same chromosome were considered for the statistical analysis. Scale bar, 5 μM. (**F**) Physical distance, in the nuclei, between probe pairs evaluated considering the genomic distance of the probes, and the genotype of the cells. Each dot in the graph represents the physical distance (measured in µm) between the hybridized probe pair. Statistical differences, ns: not significant; **** *p* < 0.0001. (**G**) Reconstruction of the loops identified in the 15q12-q13.1 region with the position of the BAC probes shown in (**A**). The size of each loop reflects the corresponding genomic size, and the genes with their transcriptional orientation are indicated in each loop. The blue symbols at the basis of the loops indicate the position of the CTCF/cohesins. Numbers 1 to 5 indicate the position of the probes used in FISH localization, and letters a, b, and c are the chromatin loops shown in Figure 1.

The degree of chromatin compaction of the chromosomal region containing *HERC2/OCA2* locus was analyzed by FISH with BAC probes (see Figure 3). More precisely, we hybridized a couple of BAC probes containing DNA sequences located at different genomic distances: a couple with a distance of 2.6 Mb between each other (probe#1, RP11-339C21; and probe#4, RP11-640H21), another distant 1.3 Mb one to each other (probe#2, RP11-299F22; and probe#3, RP11-39L5), and another couple of BACs with DNA sequences partially overlapping (probe#4, RP11-640H21; and probe#5, RP11-142A11) (Figure 3A). In regard to the location of the BAC probes in respect to the identified loops, the probes #1, #4, and #5 are located close to the loop borders; probes #2 and #3 are located far from the loop borders (Figure 3).

The FISH results for the metaphase chromosomes highlighted the specificity of each probe via a visualization of the hybridization signals only in the two chromosomes 15 (Figure 3B). Four fluorescence spots were detected in the cell nuclei due to the two hybridized BAC probes (Figure 3C–E).

To assess the degree of chromatin decondensation/compaction, the hybridized cell nuclei were examined by measuring the physical distance between green and red signals from probes located on the same chromosome. Over two hundred nuclei for each pair of probes, randomly chosen from those displaying clear hybridization signals on distinct homologous chromosomes, were analyzed by recording the distance between the two probes. Subsequently, statistical analyses, detailed in the Materials and Methods section, were conducted for the three pairs of probes in human lymphocyte nuclei with genotypes AA, AG, or GG at the SNP rs12913832 (Figure 3F).

The results were divided into two categories: one included data on the distance between various pairs of probes in cells with the same genotype, while the other included data on the distance between the same pair of probes in cells with differing genotypes. In the first case, physical distances in the cell nuclei between pairs of probes with genomic distances ranging from 0 Mb to 1.3 Mb to 2.6 Mb were measured. These pairs of probes were selected based on their positioning within the loops identified in the 15q12-q13.1 region: partially overlapping loci, loci situated at the base of the loops, and loci positioned within the chromatin loops. The results obtained from cells with the AA genotype revealed that the greatest distance (2.2 µm) was associated with the pair of probes #2-#3 positioned 1.3 Mb apart, located within the two largest chromatin loops. Conversely, the pair of probes #1#4 positioned 2.6 Mb apart but located at the base of the chromatin loops exhibited a smaller distance (1.3 µm), which was statistically highly different from the results obtained with probes positioned 1.3 Mb apart (*p* < 0.0001). As expected, the partially overlapping probes were observed at the shortest distance (0.7 µm). Similar findings were observed in cells with the AG genotype, where the probes #2#3 and #1#4, showed a distance of 1.8 and 1.4 µm, respectively. However, in cells with the GG genotype, the distance between loci positioned 1.3 (probes #2#3) and 2.6 (probes #1#4) Mb apart did not show statistical differences, with the evaluated distances being 0.9 and 1.0 µm, respectively (Figure 3F).

In the second case, it was observed that overlapping loci (probe#4 and probe#5) exhibited similar physical distances in cells with the three different genotypes, as expected. However, pairs of loci situated 1.3 and 2.6 Mb apart along the genomic DNA (probe #2#3 and probe#1#4, respectively) showed a greater physical distance in the nucleus of cells with AA and AG genotypes in respect to the distance measured in the cells with the GG genotype. The distance differences were statistically highly significant (*p* < 0.0001) (Figure 3F). Moreover, the larger difference between the distances obtained in cells with the AA/AG genotype compared to those obtained in cells with the GG genotype was detected with the probe pair #2#3 compared to the probe pair #1#4. 

The results obtained using various probes in cells with identical genotypes align with the loop structure identified in the 15q12-q13.1 region, demonstrating a lesser physical distance between loci that are genomically more distant (probe pair #1#4). Moreover, findings with the same pair of probes but in cells with different genotypes highlight the alternative effect on the chromatin loop organization of the A allele in respect to the G allele.

### 2.3. Contact Map of the Mouse 7qB5 Region Syntenic to the Human 15q12-q13.1 Bands

**Figure 4:**
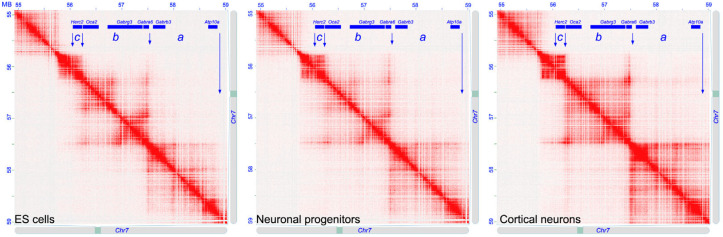
Contact map of mouse syntenic region to the 15q12-q13.1 human chromosomal bands. Heat map of the mouse chromosome 7qB5 region containing the *HERC2*, *OCA2*, *GARBG3*, *GARBA5*, *GARBB3*, and *ATP10A* genes in the ES cells, neuronal progenitors, and cortical neurons, respectively. The position of the genes is indicated at the top of each panel. The arrows show the landmarks of the chromatin loops. The letters a, b, and c indicate the human syntenic region shown in Figure 1. Hi-C data from [65].

We analyzed the heat map of the syntenic region of the 15q12-q13.1 bands in the mouse (Figure 4), and more precisely in three cell lines related to neuronal development: embryonic stem (ES) cells, neuronal progenitors, and cortical neurons (data from [65]). In these cell lines, we identified the same three chromatin loops as in the human lymphocytes. This indicates a general evolutionary conservation of the analyzed chromatin loops in a different species, which is also conserved during cell differentiation.

### 2.4. Genomic Organization of HERC2/OCA2 Region in Different Vertebrate Species

**Figure 5:**
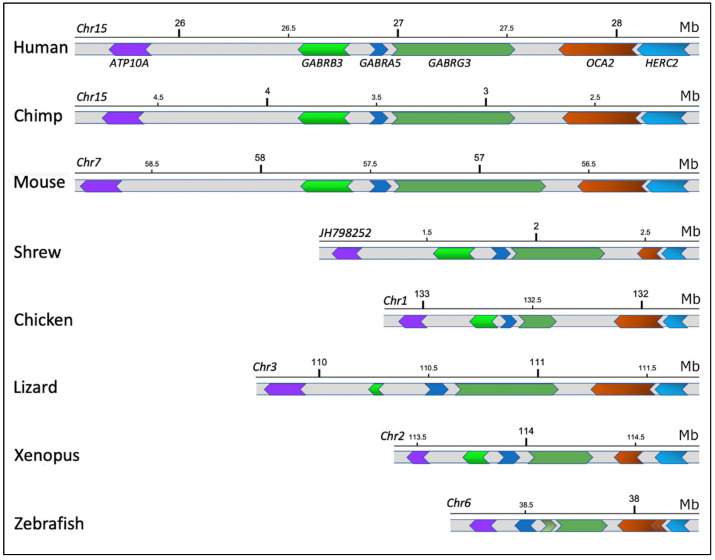
Evolutionary conservation of *HERC2/OCA2* genomic region. *ATP10A*, *GABRB3*, *GABRA5*, *GABRG3*, *OCA2*, and *HERC2* genes are indicated in the human genomic segment of the chromosome 15. In the other species, the same colors identify the orthologous genes. The chromosome of each species containing the indicated region is indicated on the left side. Scale bars (Mb: Mega base pairs) indicate the position along the chromosome, as obtained by the UCSC genome browser (https://genome.ucsc.edu, accessed on 15 February 2024). Human, *Homo sapiens*; chimp, *Pan troglodytes*; mouse, *Mus musculus*; shrew, *Sorex araneus*; chicken, *Gallus gallus*; lizard, *Anolis carolinensis*; xenopus, *Xenopus tropicalis*; zebrafish, *Danio rerio*. Data from each genomic region are presented in Supplementary Tables S1 and S2.

To evaluate the level of evolutionary conservation of the human chromosomal region 15q12-q13.1 organization, the genomes of various Vertebrate species were analyzed. The orthologous genes to the human genes *HERC2*, *OCA2*, *GARBG3*, *GARBA5*, *GARBB3*, and *ATP10A* were identified, and the size and structure of exons/introns, direction of transcription, and reciprocal positions were detected. The results demonstrated the high conservation rate of the gene organization and, in particular, of the genes *OCA2* and *HERC2*. These two genes are organized in a head-to-tail fashion, with the 3′-end of the *HERC2* gene positioned upstream of the *OCA2* gene in all species analyzed. The other genes are also present with the same orientation and position (Figure 5).

## 3. Discussion

Eye color is mainly determined by a well-known pigmentation gene, *OCA2*, which is involved on the regulation of the amount, type, and distribution of melanin present in the iris [66,67]. In fact, specific variants in this gene are associated with a greater or lesser production of melanin, directly responsible for variation in iris pigmentation, resulting in darker or lighter eyes [56].

However, despite the absence of a known biological role in pigmentation for the product of the *HERC2* gene where the SNP rs12913832 is located in intron 86, a functional role has been suggested for this SNP. The region encompassing rs12913832 is highly conserved across species [15,56] and is located within a distal regulatory element that modulates the transcription of the *OCA2* gene [4,13,14,68]. To shed light on the role of SNP rs12913832 in the organization of the *HERC2/OCA2* locus in the nucleus and its impact on *OCA2* expression, this study aimed to achieve a comprehensive understanding of the nuclear and functional organization of the 15q12-13.1 region by integrating data from various experimental techniques and public genomic data resources, which also revealed the high level of evolutionary conservation of this region in the Vertebrate genome.

The TADs organization of the 15q12-q13.1 region obtained from the Hi-C data indicated the presence of three chromatin loops, with two larger loops and the third, the smallest, containing only the *HERC2* gene. The other genes are *ATP10* and *GABRB3*, located in the larger more centromeric loop; and the *GABRA5*, *GABRG3*, and *OCA2* genes, located in the intermediate loop (Figure 1). This loop organization is largely conserved in the different cell types considered here, not only in human but also in mouse cells. Moreover, considering the high level of evolutionary conservation of gene organization of this region among Vertebrates, we can suppose that the TAD organization observed in the human cells should also be present in other Vertebrate species. This organization likely has functional or structural relevance conserved during evolution.

The organization of the 15q12-q13.1 region into three chromatin loops was visualized with Hi-C data (Figure 1) and experimentally demonstrated through in situ hybridization with probes located in this region (Figure 3). The physical distance between loci within this region, in the nuclei of human lymphocytes, is consistent with the three-loop structure described here. Specifically, we demonstrated that loci that are farther apart in the genome (2.6 Mb) but located at the base of chromatin loops have a smaller physical distance in the nucleus compared to loci that are closer in the genome (1.3 Mb), but positioned on the outer part of the loop. Thus, probes #1#4, which are 2.6 Mb apart, are positioned at a distance of 1.3 µm in the nucleus, while probes #2#3, which are 1.3 Mb apart, are positioned at a distance of 2.2 µm. This indicates a closer physical proximity between the first pair of probes compared to the second. This situation was observed in cells with the AA genotype. In cells with the heterozygous AG genotype, the result is similar, although the difference between the two measurements is less pronounced. Specifically, in cells with the AA genotype, the two measurements (distance of probes #2#3 vs. probes #1#4) differ by approximately 0.9 µm, while in cells with the AG genotype, the difference is about 0.4 µm. Moreover, the pair of probes #2#3 in the cells with GG genotype shows a much smaller mutual distance compared to cells with the AA or AG genotype. Specifically, the probes #2#3 exhibit a mutual distance of about 0.9 µm, a value not statistically different from the pair of probes #1#4 in the same GG cells. This indicates a positive effect of the A allele on the opening of the chromatin loop. Indeed, in cells with AA or AG genotypes, the distance between the pair of probes #2#3 is much larger than in cells with the GG genotype.

If we consider the distance between a specific pair of probes in cells with different genotypes, we clearly observe that, in the AA and AG genotypes, the distance between the two loci is always much larger than that obtained in cells with the GG genotype, except for the overlapping probes (probe pair #4#5), which show, as expected, similar distances in the nuclei. This is particularly evident with the pair of probes located within the chromatin loops (probe pair #2#3), while it is less pronounced (though statistically significant) with the pair of probes located at the base of the chromatin loops (probe pair #1#4). It is noteworthy that this reorganization of the chromatin loops according to cell genotype is associated with a variation in a single nucleotide, which, based on the data obtained, should be capable of inducing a reshaping of the loop structure of the chromatin in the 15q12-q13.1 chromosomal region.

These findings suggest that the compaction level of loops in the 15q12-q13.1 region, particularly those involving *OCA2* and *HERC2* genes, varies depending on cell genotype, specifically related to SNP rs12913832. This indicates that single-nucleotide genetic variations can influence loop organization. Such variations can affect gene expression and regulation, as chromatin looping brings distant genomic regions closer, impacting gene accessibility to regulatory elements and expression patterns. Differences in loop structure and compaction among cells with different genotypes suggest a potential mechanism for how genetic variations influence gene regulation. This mechanism, which involves the action of an SNP located in an intron of a gene different from the one whose regulation has an effect, could be much more represented in the human genome than we currently know.

Our research underscores the significance of studying both coding and non-coding regions to fully appreciate the regulatory mechanisms at play. The combined use of Hi-C and FISH allowed us to map the 3D genome architecture and directly observe chromatin interactions, respectively. This integrated approach elucidates the significant impact of the rs12913832 genotype on chromatin compaction of the *OCA2* gene, thereby influencing eye color pigmentation, and contributes to a more precise understanding of genome organization and its impact on gene regulation, which is essential for accurate phenotypic predictions.

It is crucial to emphasize that many diagnoses of multigenic diseases rely on results obtained through high-throughput sequencing methods, such as whole-exome sequencing (WES). With this approach, exons of genes were analyzed, along with a few nucleotides located in introns near the splicing sites. While WES is regarded as a powerful diagnostic tool, some relevant mutations, potentially not yet identified, may exist in intronic regions distant from the splicing sites’ boundaries, which cannot be identified by WES alone. Therefore, in the absence of detectable mutations using the WES system, it is advisable to conduct a comprehensive analysis that encompasses not only exonic sequences but also intronic.

## 4. Materials and Methods

### 4.1. Cell Cultures, Preparation of Chromosomes and Nuclei, and Genotype Identification

Human lymphocytes were obtained from whole peripheral blood cultured for 72 h in RPMI 1640 supplemented with 20% FBS, 1% penicillin/streptomycin, 1% L-glutamine, 3% PHA at 37 °C, and 5% CO2. Moreover, stimulation was performed by addition of phytohemagglutinin (PHA) to the culture. Metaphase chromosomes and nuclei were then prepared using a hypotonic solution (KCl 0.075M) according to standard cytogenetic procedures and methanol/acetic acid (3:1) to fix samples. The use of lymphocytes from healthy volunteers was in accordance with the ethical standards of the institutional and/or national research committees and with the 1964 Helsinki declaration and its later amendments or comparable ethical standards. 

The genotyping was carried out to obtain the DNA sequence of rs12913832. The experiments were performed with the TaqMan SNP Genotyping Assay C_30724404_10 (Applied Biosystem, Vilniusm, Lithuania) commercially available from the manufacturer, and a total of 2 µL genomic DNA extract was amplified in 20 µL qPCR reaction with 2X TaqMan. 

Genotyping Master Mix (4371353, Applied Biosystem, Vilniusm, Lithuania) and 2OX assay TaqMan Mix. DNA was dispensed in 48-well plates by qPCR (with StepOne instrument from Applied Biosystems, Foster City, CA, USA), and the PCR program was 60 °C for 30 s, 95 °C for 10 min, 40 cycles of 95 °C for 15 s, and 60 °C for 1 min.

### 4.2. Hi-C Dataset and Analysis

The Hi-C data refer to those described by [64] in which the resolution of the regions of contact reaches very high levels (up to 1 Kb). The datasets used were those obtained through the in situ combined procedure, and the identification of the number of contacts of the chromosomal regions analyzed was obtained using the Juicebox software v. 1.11.08, purposely designed by Dr. Lieberman Aiden. The heat maps, showing the interaction between the chromosomal regions analyzed, were generated in both low resolution (500 kb) and high resolution (5 kb). The genomic characteristics considered were the chromosomal bands, with their GC level, obtained from [31]; the genes included; and the epigenetic chromatin characteristics from Genome Browser of UCSC. In particular, in order to have data comparable with the heat maps, the human genome sequence of Feb 2009 (GRCh37/hg19) and the mouse one of December 2011 (GRCm38/mm10) were used. For the evolutionary conservation, the gene organization in 15q12-13.1 bands was carried out using the database made available by the University of California Santa Cruz through the Genome Browser Gateway, using the most recently released genomes. 

### 4.3. DNA Probes, In Situ Hybridization and Detection

FISH experiments were performed using specific BAC probes with human DNA from loci of the chromosomes 15 (15q12-q13.1 band) (Table 1 and Figure 3). DNA probes were extracted using a commercial kit NucleoSpin Plasmid (Macherey-Nagel, Duren, Germany), digoxigenin, or biotin-labelled by nick translation (Roche, Mannheim, Germany) and hybridized, as previously described [31]. Detection was carried out using rhodamine-conjugated avidin (for biotin-labelled probes), and anti-digoxigenin secondary antibody conjugated with fluorescein (for digoxigenin-labelled probes). Hybridization signals on metaphase chromosomes and interphase nuclei were analyzed using a Nikon ECLIPSE N*i*-E fluorescence microscope (Nikon corporation, Tokyo, Japan) and captured with a Nikon DS-Qi2 camera (Nikon corporation, Tokyo, Japan). Images were recorded using Nikon NIS-Elements Imaging Software v. 5.20.01 (Nikon corporation, Tokyo, Japan). Cell nuclei were then analyzed for the evaluation of radial location of each probe and to measure the distances between the two probes.

### 4.4. Physical Distance between Loci in the Interphase Nucleus

The physical distance between two probes in a single cell nucleus were measured with Nikon NIS-Elements Imaging Software v. 5.20.01 (Nikon corporation, Tokyo, Japan). Then, the evaluation of the physical distance was obtained via a statistical analysis of the data obtained from at least 200 nuclei that were randomly selected. More precisely, the statistical analyses were carried out considering the 40 measurements with the highest values. This is because, in the 2D analysis, the large numbers of a couple of signals, due to the hybridized pairs of BAC, are not in the same orthogonal plane in respect to the observer; thus, the relative distance is not the real distance (see Supplementary Figure S1). So, we considered only the larger sets of distances, namely the case of the BACs in their maximum point of distance. The statistical differences between pairs of groups were determined by performing Student’s *t*-test, and the level of significance was set at *p* < 0.05 (*), *p* < 0.01 (**), *p* < 0.001 (***), and *p* < 0.0001 (****).

## 5. Conclusions

To enhance our understanding of predicting complex phenotypic traits, such as eye color, it is crucial to delve deeper into the molecular mechanisms related to SNPs situated in non-coding regions, exemplified by the SNP rs12913832 in the *HERC2* gene. While *HERC2*’s product does not directly influence melanin production, its impact on *OCA2* expression is significant, indicating the pivotal role of a single-nucleotide polymorphism in modulating gene expression. Previous studies have demonstrated the interaction between the SNP rs12913832 and the *OCA2* promoter, resulting in the formation of a chromatin loop. Notably, the nucleotides adjacent to this SNP in intron 86 of *HERC2* are evolutionarily widely conserved in mammals [12,13,54].

Here, we analyze the chromatin loop organization over a larger region (~3 Mb), examining the compaction/decondensation levels based on the rs12913832 genotype, using interphase-FISH experiments with BAC probes, an approach not previously described. By combining the strengths of FISH and Hi-C, we provided compelling evidence for the role of the intronic SNP rs12913832 in chromatin loops that exhibit varying degrees of compaction depending on the genotype. This indicates a genotype-dependent long-range impact on chromatin organization. This integrated approach allows us to uncover the broader chromatin architecture and emphasize the importance of intronic sequences in genetic studies of complex traits like eye color pigmentation.

This mechanism assumes great significance within the scrutinized region, given the considerable evolutionary conservation observed in the genomic organization of *HERC2* and *OCA2* genes, as well as other contiguous genes. We highlighted the evolutionary conservation over a region of approximately 3 Mb adjacent to *OCA2*, containing six genes, across all classes of Vertebrates. Previous work, however, evaluated the conservation of only the nucleotide region around the SNP in mammals [12,54]. This indicates that, in addition to the SNP in question, the strongly conserved organization of genes and their transcriptional orientation likely play a relevant role, acting as a superstructure that includes the abovementioned SNP.

While diagnostic procedures employing massive exon sequencing (whole-exome sequencing) significantly aid in identifying SNP genotypes linked to multigenic disorders, caution is warranted in interpreting results due to the absence of data on intronic sequences. The evolutionary conservation of gene organization, as seen in the 15q12-q13.1 region examined here, underscores the critical role of chromatin loop structures containing multiple genes, where an intronic SNP can play a central role in genome organization within interphase nuclei and in the regulation of the genes they contain.

An integrated investigation utilizing various experimental methodologies (genomic analyses, Hi-C, FISH, etc.) to study genome organization in interphase nuclei facilitates a deeper comprehension of the role of chromatin organization in shaping phenotypic traits. This not only aids in refining the diagnostic aspects of specific SNPs but also contributes to a more precise and reliable phenotypic prediction system, particularly in forensic applications. Future perspectives can include inducing mutations in SNP rs12913832 in melanocytic cells to further explore these associations between the rs12913832 genotype and chromatin compaction/decondensation levels.

## Figures and Tables

**Table 1:** BAC probes used in the present study.

Probe	Chromosome Band	Position (bp)	Genomic Size (bp)
RP11-339C21	15q12	25,540,816–25,708,423	167,608
RP11-299F22	15q12	26,450,149–26,643,939	193,791
RP11-39L5	15q12	27,604,108–27,745,105	140,998
RP11-640H21	15q13.1	27,894,428–28,091,240	196,813
RP11-142A11	15q13.1	28,042,426–28,192,499	150,074

## Data Availability

Data are contained within the article and in Supplementary Materials.

## Supplementary Materials

The supporting information can be downloaded at https://www.mdpi.com/article/10.3390/ijms25126602/s1.
