# Supplementary material for: The Chromatin Organization Close to SNP rs12913832, Involved in Eye Color Variation, Is Evolutionary Conserved in Vertebrates
Source: Int J Mol Sci. 2024 Jun 15;25(12):6602. doi: 10.3390/ijms25126602 (PMC11204186; doi:10.3390/ijms25126602)
Supplement: Supplementary file 1 [file ijms-25-06602-s001.zip › Supplementary file2.pdf]

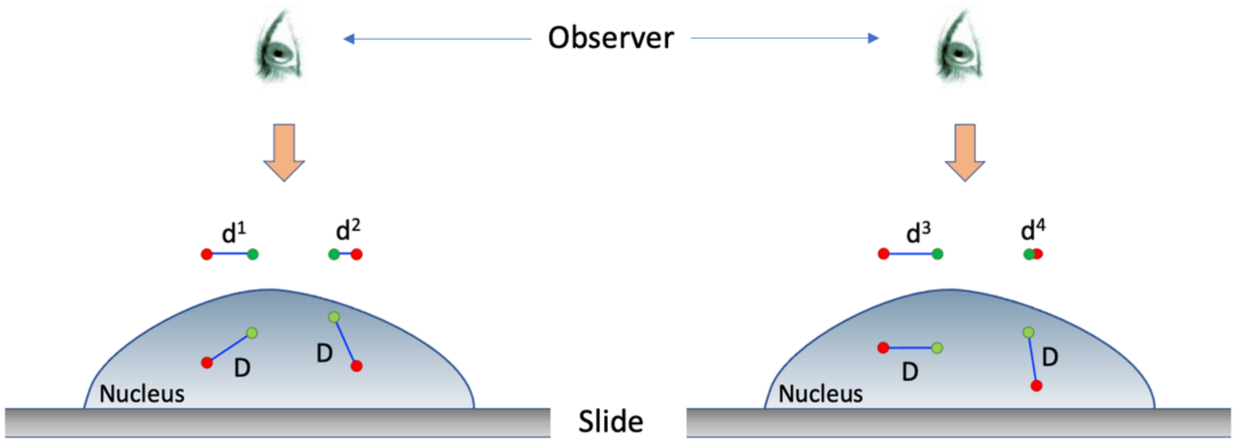

Figure S1. Evaluation of the physical distance between two BAC probes by *in situ* hybridization. BAC probes containing human DNA from the region of interest, after *in situ* hybridization and detection with different fluorochromes (Fluorescein-green, and Rhodamine-red) in the cell nuclei, were recorded to measure the distance between probe pairs. This was done by using the Nikon ECLIPSE N*i*-E fluorescence microscope (Nikon corporation, Tokyo, Japan), equipped with a Nikon DS-Qi2 camera (Nikon corporation, Tokyo, Japan). Cell nuclei were recorded using Nikon NIS-Elements Imaging Software (Nikon corporation, Tokyo, Japan). The images illustrate examples of detected distances (d1, d2, d3, d4) in a 2D image record, with reference to the actual distance indicated by D in the nucleus cartoon. The large number of detected distances are smaller than the real distance D, depending on the position of the two fluorescent spots one to each other and respect to the observer. Only in the case the two spots are in the same plane, orthogonal to the view of observation, the distance is the same of the real D, as in the case of d3 distance.
